# Validation of reference genes for quantitative real-time PCR during leaf and flower development in *Petunia hybrida*

**DOI:** 10.1186/1471-2229-10-4

**Published:** 2010-01-07

**Authors:** Izaskun Mallona, Sandra Lischewski, Julia Weiss, Bettina Hause, Marcos Egea-Cortines

**Affiliations:** 1 Genetics, Instituto de Biotecnología Vegetal, Universidad Politécnica de Cartagena (UPCT), 30203 Cartagena, Spain; 2 Leibniz-Institut für Pflanzenbiochemie, Weinberg 3, PO Box 110432, D-06120 Halle (Saale), Germany

## Abstract

**Background:**

Identification of genes with invariant levels of gene expression is a prerequisite for validating transcriptomic changes accompanying development. Ideally expression of these genes should be independent of the morphogenetic process or environmental condition tested as well as the methods used for RNA purification and analysis.

**Results:**

In an effort to identify endogenous genes meeting these criteria nine reference genes (RG) were tested in two Petunia lines (Mitchell and V30). Growth conditions differed in Mitchell and V30, and different methods were used for RNA isolation and analysis. Four different software tools were employed to analyze the data. We merged the four outputs by means of a non-weighted unsupervised rank aggregation method. The genes identified as optimal for transcriptomic analysis of Mitchell and V30 were *EF1α *in Mitchell and *CYP *in V30, whereas the least suitable gene was *GAPDH *in both lines.

**Conclusions:**

The least adequate gene turned out to be *GAPDH *indicating that it should be rejected as reference gene in Petunia. The absence of correspondence of the best-suited genes suggests that assessing reference gene stability is needed when performing normalization of data from transcriptomic analysis of flower and leaf development.

## Background

The general aims of transcriptomic analysis are identification of genes differentially expressed and measurement of the relative levels of their transcripts. Transcriptomic analysis like that relying on microarray techniques reveals an underlying expression dynamic that changes between tissues and over time [1]. Results must then be validated by other means in order to obtain robust data that will support working hypotheses directed at a better understanding of development or environmental responsiveness. Since the advent of quantitative PCR, it has become the method of choice to validate gene expression data. However, data obtained by qPCR can be strongly affected by the properties of the starting material, RNA extraction procedures, and cDNA synthesis. Therefore, relative quantification procedures require comparison of the gene of interest to an internal control, based on a normalization factor derived from one or more genes that can be argued to be equally active in the relevant cell types. This requires the previous identification of such genes, which can then be reliably used to normalise relative expression of genes of interest.

Identification of candidate genes useful for normalization has become a major task, as it has been shown that normalization errors are probably the most common mistake, resulting in significant artefacts that can lead to erroneous conclusions [2]. Several software tools have been developed to compute relative levels of specific transcripts (commonly referred to as 'gene expression', although obviously transcript stability is also an important factor contributing to transcript levels) based on group-wise comparisons between a gene of interest and another endogenous gene [3]. However identification of genes with stable patterns of gene expression requires pairwise testing of several genes with each other. Among the software programs developed toward this end are geNorm [4], BestKeeper [5], NormFinder [6] or qBasePlus [7]. The programs geNorm and qBasePlus use pairwise comparisons and geometric averaging across a matrix of reference genes. qBasePlus also calculates a coefficient of variation (CV) for each gene as a stability measurement. BestKeeper uses pairwise correlation analysis of each internal gene to an optimal normalization factor that merges data from all of them. Finally, NormFinder fits data to a mathematical model, which allows comparison of intra- and intergroup variation and calculation of expression stability.

Using the programs described above researchers have identified genes suitable for use as normalization controls in Arabidopsis [8], rice [9], potato leaves [10], the parasitic plant *Orobanche ramosa *[11], *Brachypodium distachyon *[12] and grape [13]. In the Solanaceae, candidate genes for normalization have been determined based on EST abundance [14], and qPCR followed by statistical analysis using the tools described above have been reported [15].

A feature shared amongst these studies, and a large number of additional publications describing human, animal and plant systems, is the identification of genes specific for a certain tissue, developmental stage or environmental condition. This is a logical experimental design, as individual research programs tend to be focused, and the number of appropriate genes can be expected to be inversely related to the number of cell types or conditions under investigation. Recent studies that included different cultivars of soybean [16], underscore how the characteristics of the plant and the types of organs studied must drive the experimental approach to transcriptomic analysis.

The garden Petunia (*Petunia hybrida*) has been extensively used as a model for developmental biology [17,18]. Amongst the inbred Petunia lines used in research, the white-flowered Mitchell [19], also known as W115, is routinely exploited for transformation and scent studies [20-22]. The genetics of flower pigmentation has been intensively studied in lines such as V30 [23]. Mitchell and V30 are genetically dissimilar, as demonstrated in mapping studies, and vary in a number of other ways, including growth habit and amenability to propagation in culture. Here we have used multiple developmental stages of flowers and leaves of these two Petunia lines to identify genes that show reliable robustness as candidates for use in normalization of relative transcript abundance. The experiments were carried out in two different laboratories, with different PCR machines and different purification and amplification conditions. We found that the final shortlist of valuable genes was different between lines suggesting the necessity of performing reference gene stability measurements as part of the experimental design where differences in gene expression in Petunia is tested.

## Results

(1948 w)

### Petunia lines, developmental stages and selection of genes for normalization

**Figure 1:**
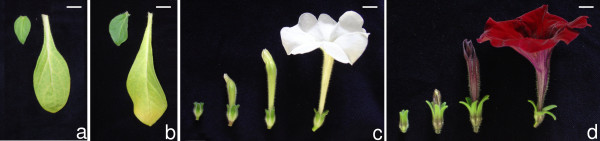
**Developmental stages of leaves and flowers used for RNA extractions**. Representative photographs of leaves and flowers of *Petunia hybrida *lines Mitchell (a, c) and V30 (b, d) are shown. The leaf stages are young, small leaf (leaf of the left in a, b) and fully expanded leaf (leaf of the right in a, b). Flowers at four different developmental stages are shown (c, d). From left to right they range from young flower bud (stage A, 1-1.5 cm), over-elongated bud (stage B, 2.5-3 cm) and pre-anthesis (stage C, 3.5-4.5 cm) to fully developed flower (stage D, open flower).

Two very different Petunia lines were used for the analyses. Mitchell, also known as W115, is a doubled haploid line obtained from anther culture of an interspecific Petunia hybrid [19]; it is characterized by vigorous growth, exceptional fertility, strong fragrance and white flowers. V30 is an inbred line of modest growth habit and fertility featuring deep purple petals and pollen. From each line we harvested flowers representing four developmental stages, from young flower buds to open flowers shortly before anthesis, and two leaf developmental stages, young and full-sized (Figure 1).

Potentially useful RG were selected based on review of the relevant literature, from which we identified genes previously used for normalization or routinely used as controls for northern blots or RT-PCR. From the original list we developed a short list of nine, including genes encoding *Actin-11 (ACT*), *Cyclophilin-2 *(*CYP*)[10], *Elongation factor 1α *(*EF1α*), *Ubiquitin *(*UBQ*) *Glyceraldehyde-3-phosphate dehydrogenase *(*GAPDH*), *GTP-binding protein RAN1 *(*RAN1*), *SAND protein *(*SAND*) [8,24,25], *Ribosomal protein S13 *(*RPS13*) [6] and *b-Tubulin *6 (*TUB*) [26] (Table 1). The products of these genes are associated with a wide variety of biological functions. Moreover, these genes are described as not co-regulated, a prerequisite for using one of the algorithms to identify stably expressed genes (geNorm) reliably [4].

**Table 1:** Genes, primers and amplicon characteristics

**Gene name**	**Molecular function**	**Accesion**	**Tblastx e-value**	**Primer sequences (forward/reverse)**	**Length (bp)**	**Efficiency**
*ACT*	Actin 11	SGN-U208507 (At3 g12110.1)	2e-110	TGCACTCCCACATGCTATCCT/TCAGCCGAAGTGGTGAAAGAG	114	1.75 ± 0.07
*CYP*	Cyclophilin	SGN-U207595 (At2 g21130.1)	1.9e-75	AGGCTCATCATTCCACCGTGT/TCATCTGCGAACTTAGCACCG	111	1.64 ± 0.10
*EF1α*	Elongation factor 1-alpha	SGN-U207468 (At5 g60390.1)	0	CCTGGTCAAATTGGAAACGG/CAGATCGCCTGTCAATCTTGG	103	1.62 ± 0.08
*GAPDH*	Glyceraldehyde-3-phosphate dehydrogenase	SGN-U209515 (At1 g42970.1)	9.2e-79	AACAACTCACTCCTACACCGG/GGTAGCACTAGAGACACAGCCTT	135	1.83 ± 0.09
*RPS13*	Ribosomal protein S13	SGN-U208260 (At4 g00100.1)	4e-77	CAGGCAGGTTAAGGCAAAGC/CTAGCAAGGTACAGAAACGGC	114	1.70 ± 0.04
*RAN1*	GTP-binding nuclear protein	SGN-U207968 (At5 g20010.1)	1e-119	AAGCTCCCACCTGTCTGGAAA/AACAGATTGCCGGAAGCCA	103	1.71 ± 0.07
*SAND*	*SAND *family protein	SGN-U210443 (At2 g28390.1)	8.2e-76	CTTACGACGAGTTCAGATGCC/TAAGTCCTCAACACGCATGC	135	1.61 ± 0.12
*TUB*	Tubulin beta-6 chain	SGN-U207876 (At5 g12250.1)	6e-147	TGGAAACTCAACCTCCATCCA/TTTCGTCCATTCCTTCACCTG	114	1.61 ± 0.05
*UBQ*	Polyubiquitin	SGN-U207515 (At4 g02890.2)	8e-107	TGGAGGATGGAAGGACTTTGG/CAGGACGACAACAAGCAACAG	153	1.67 ± 0.02

Selected candidate reference genes accessions are shown as identifiers of Solanaceae Genomics Network (SGN) and Arabidopsis TAIR databases (in brackets). Homologous Arabidopsis genes were determined on the basis of tblastx e-values

### Strategy for data mining and statistical analysis

**Figure 2:**
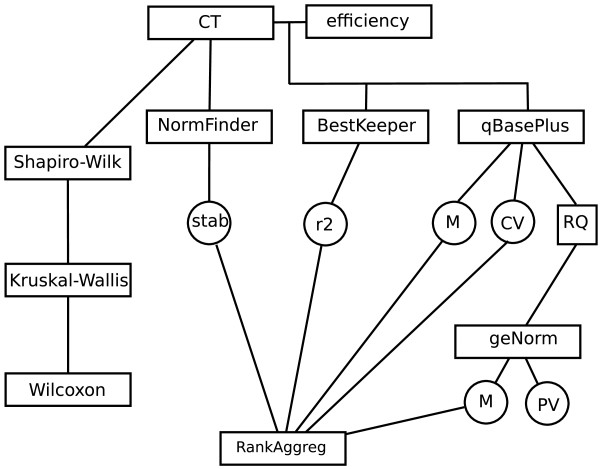
**Data analysis flow chart**. CT (cycle threshold) values were calculated using different thresholds depending on the variety. Efficiency value taken for line Mitchell was 2; for line V30, there was one value for each tube. Circles indicate statistical results to be merged with RankAggreg (Pihur 2009). Relative quantities (RQ) were scaled to the sample with lowest CT value (flower stage C). CT data were checked for normality (Shapiro-Wilk test) and, due to non-normality, they were analysed by non-parametrical tests (Kruskal and Wallis). Since CT values showed non-equal distributions according to the organ from which RNA was extracted, they were further tested using pairwise Wilcoxon tests with Bonferroni's correction with the aim of solving pairwise significant variations. A significance threshold of 0.05 was used. Abbreviations: PV, pairwise variation; M, classical stability value; stab, NormFinder stability value; CV, variation coefficient; r2, determination coefficient - regression to BestKeeper; RQ, relative quantities.

The genes described above were selected to test for stability of transcript levels through leaf and flower development in two Petunia lines, Mitchell and V30. As the aim of the present work is to find if we could obtain a similar rank of genes irrespective of the Petunia line, growth conditions or sample processing, we developed all the data mining procedures separately for each line. Cycle threshold (CT) values were determined and expression stability, i.e., the constancy of transcript levels, ranked. As a strategy for calculating relative expression quantities (RQ) we applied the qBasePlus software, taking into account for each reaction its specific PCR efficiency. Rescaling of normalized quantities employed the sample with the lowest CT value (see materials and methods and Figure 2). With qBasePlus we measured expression stability (M values) and coefficients of variation (CV values). Relative quantities were transferred to geNorm for computing M stability values. It is worth noting that the procedure for computing M values differs between geNorm and qBasePlus. Finally, we used the combined stability measurements produced by geNorm, NormFinder, BestKeeper and qBasePlus to establish a consensus rank of genes by applying RankAggreg [27]. The input to this statistical package was a matrix of rank-ordered genes according to the different stability measurements previously computed. RankAggreg calculated Spearman footrule distances and the software reformatted this distance matrix into an ordered list that matched each inital order as closely as possible This consensus rank list was obtained by means of the Cross-Entropy Monte Carlo algorithm present in the software.

### CT values and variability between organs and developmental stages in Mitchell and V30

Real-time PCR reactions were performed on the six cDNA samples obtained from each Petunia line with the nine primer pairs representing the candidate RG. In order to assess run reliability non-template controls were added and three technical repetitions were included for each biological replicate. CT values were defined as the number of cycles required for normalized fluorescence to reach a manually set threshold of 20% total fluorescence. Product melting analysis and/or gel electrophoresis allowed for the discarding of non-specific products. Moreover, we considered only CT technical repetitions differing by less than one cycle.

**Figure 3:**
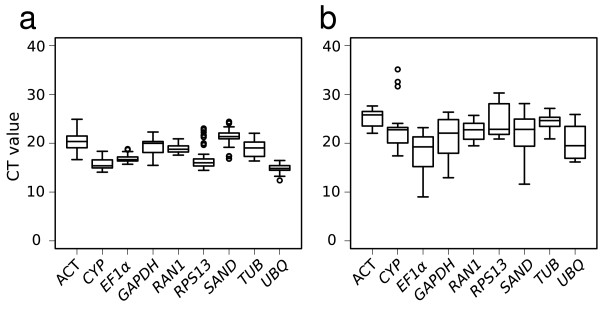
**Expression profiling of reference genes in different organs and Petunia lines**. CT values are inversely proportional to the amount of template. Global expression levels (CT values) in the different lines tested are shown as 25th and 75th quantiles (horizontal lines), median (emphasized horizontal line) and whiskers. Whiskers go from the minimal to maximal value or, if the distance from the first quartile to the minimum value is more than 1.5 times the interquartile range (IQR), from the smallest value included within the IQR to the first quartile. Circles indicate outliers, the values smaller or larger than 1.5 times the IQR.

The CT values obtained for all the genes under study differed between the two Petunia lines (Figure 3). The range of values was consistently narrower in Mitchell than in V30. This could indicate that gene expression in general is less variable in Mitchell than in V30, however these data correspond to averages derived from all the samples and further analysis showed that in fact V30 exhibited more constant levels of tested transcripts at the single organ level or developmental stage (see below).

For Mitchell samples *UBQ *was the most highly expressed gene overall, with a CT of 14.8, and *SAND *the lowest, with a CT of 21.2. In contrast, the highest and lowest expressed genes in V30 were *EF1α *and *ACT*, with CTs of 18.3 and 25.1, respectively.

Analysis of variance of CT values between organs was performed separately for Mitchell and V30 samples. Since CT values were not normally distributed, we calculated Kruskall-Wallis and a *post-hoc *Pairwise Rank Sum Wilcoxon test, both non-parametrical, using a Bonferroni correction and a significance cut-off of 0.05. In Mitchell the genes *RAN1*, *RPS13 *and *UBQ *showed significant differences in transcript levels between developmental stages (Additional file 1). *RAN1 *transcript levels differed significantly between leaf A and flowers C and D, *RPS13 *differed in flower D from the rest of floral stages analysed, and *UBQ *transcript levels differed significantly between leaf A and flower D. For V30, the overall CT variability was higher than that seen in Mitchell; in fact, expression of all the genes analysed showed significant differences between one or more sets of organs and/or developmental stages. Expression of the genes *GAPDH *and *TUB *differed between leaves A and C, while levels of other measured transcripts were essentially the same in the two leaf stages. In contrast, during flower development, we could distinguish genes that showed two levels of significantly different CT values (*GAPDH *and *TUB*), those that showed three (*ACT*, *CYP*, *EF1α *and *RPS13*) and others that differed at each developmental stage analysed (*RAN1*, *SAND *and *UBQ*).

### Stability of gene expression in Mitchell and V30

Data from each of the two chosen Petunia lines were analyzed separately. As a first approach, we applied data as a unique population and transferred it to NormFinder, BestKeeper, geNorm and qBasePlus according to the flowchart plotted in Figure 2. In a second approach, we subdivided data into several subpopulations, corresponding to unique developmental stages (i.e., flower C or leaf A), then, piped this data into the qBasePlus and geNorm tools. The results of both sets of analyses are presented in Tables 2 and 3 and Additional files 2, 3 and 4.

**Table 2:** Optimal genes for quantification of individual and mixed organs in each Petunia line.

***Mitchell***								
**Statistic**	**Flower**					**Leaf**		
	**A**	**B**	**C**	**D**	**A+B+C+D**	**A**	**C**	**A+C**
M geNorm	*EF1α *(0.05) *RPS13 *(0.05)	*SAND *(0.14) *UBQ *(0.14)	*EF1α *(0.13) *RPS13 *(0.13)	*RAN1 *(0.08) *RPS13 *(0.08)	*RAN1 *(0.47) *SAND *(0.47)	*EF1α *(0.11) *RPS13 *(0.11)	*RAN1 *(0.14) *SAND *(0.14)	*EF1α *(0.37) *RPS13 *(0.37)
M qBasePlus	*ACT *(0.55) *RPS13 *(0.56)	*EF1α *(0.60) *RPS13 *(0.60)	*CYP *(0.60) *SAND *(0.64)	*EF1α *(0.30) *RAN1 *(0.34)	*EF1α *(0.80) *SAND *(0.82)	*RPS13 *(0.77) *EF1α *(0.78)	*SAND *(0.93) *RAN1 *(0.93)	*EF1α *(0.85) *RAN1 *(0.92)
CV qBasePlus	*ACT *(0.05) *SAND *(0.15)	*RPS13 *(0.07) *CYP *(0.25)	*CYP *(0.09) *SAND *(0.11)	*EF1α *(0.05) *TUB *(0.14)	*EF1α *(0.25) *SAND *(0.28)	*RPS13 *(0.14) *RAN1 *(0.18)	*SAND *(0.12) *RAN1 *(0.18)	*RAN1 *(0.20) *EF1α *(0.21)
Min. number	2 (0.04)	2 (0.07)	2 (0.12)	2 (0.05)	4 (0.15)	2 (0.10)	2 (0.13)	2 (0.12)
***V30***								
**Statistic**	**Flower**					**Leaf**		
	**A**	**B**	**C**	**D**	**A+B+C+D**	**A**	**C**	**A+C**
M geNorm	*RAN1 *(0.11) *UBQ *(0.11)	*TUB *(0.12) *CYP *(0.12)	*RPS13 *(0.23) *UBQ *(0.23)	*ACT *(0.02) *CYP *(0.02)	*RAN1 *(0.45) *ACT *(0.45)	*TUB *(0.07) *RAN1 *(0.07)	*RPS13 *(0.09) *TUB *(0.09)	*RAN1 *(0.20) *UBQ *(0.20)
M qBasePlus	*CYP *(0.30) *RAN1 *(0.33)	*CYP *(0.66) *TUB *(0.69)	*SAND *(0.63) *CYP *(0.63)	*RPS13 *(0.29) *EF1α *(0.30)	*ACT *(2.27) *RAN1 *(2.44)	*TUB *(0.34) *UBQ *(0.36)	*SAND *(0.27) *RPS13 *(0.29)	*UBQ *(0.49) *RPS13 *(0.51)
CV qBasePlus	*CYP *(0.05) *TUB *(0.10)	*RAN1 *(0.09) *CYP *(0.11)	*SAND *(0.10) *ACT *(0.25)	*EF1α *(0.06) *CYP *(0.06)	*ACT *(0.70) *RAN1 *(0.82)	*UBQ *(0.06) *TUB *(0.09)	*SAND *(0.03) *RPS13 *(0.06)	*UBQ *(0.14) *RPS13 *(0.16)
Min. number	2 (0.10)	2 (0.07)	2 (0.09)	2 (0.04)	NA	2 (0.04)	2 (0.03)	2 (0.07)

M values computed by geNorm and qBasePlus allow to rank optimal reference genes. For each organ and mix of organs the two top-ranked genes are shown. The number of genes required for a reliable quantification is established using a Pairwise Variation (PV) cut-off of 0.15; n is the the minimum number of control genes required NA means that no one pairwise variation was under the proposed cut-off.

**Table 3:** Gene suitability rankings for the whole dataset.

**Rank position**	**NormFinder**		**BestKeeper**		**qBasePlus M**		**qBasePlus CV**		**geNorm M**		**Consensus**	
	***Mitchell***	***V30***	***Mitchell***	***V30***	***Mitchell***	***V30***	***Mitchell***	***V30***	***Mitchell***	***V30***	***Mitchell***	***V30***
1	*EF1α*	*UBQ*	*CYP*	*CYP*	*EF1α*	*ACT*	*EF1α*	*RAN1*	*RAN1*	*RPS13*	*EF1alpha*	*CYP*
2	*CYP*	*RAN1*	*EF1α*	*EF1α*	*SAND*	*RAN1*	*SAND*	*CYP*	*SAND*	*UBQ*	*SAND*	*RAN1*
3	*RPS13*	*ACT*	*RPS13*	*ACT*	*RAN1*	*CYP*	*RPS13*	*ACT*	*UBQ*	*RAN1*	*RPS13*	*ACT*
4	*UBQ*	*GAPDH*	*ACT*	*SAND*	*RPS13*	*TUB*	*RAN1*	*TUB*	*EF1alpha*	*CYP*	*RAN1*	*UBQ*
5	*ACT*	*RPS13*	*UBQ*	*UBQ*	*CYP*	*RPS13*	*CYP*	*RPS13*	*RPS13*	*ACT*	*CYP*	*RPS13*
6	*SAND*	*SAND*	*TUB*	*GAPDH*	*UBQ*	*UBQ*	*UBQ*	*UBQ*	*CYP*	*TUB*	*UBQ*	*TUB*
7	*TUB*	*EF1alpha*	*SAND*	*RPS13*	*TUB*	*EF1alpha*	*TUB*	*EF1alpha*	*TUB*	*EF1alpha*	*TUB*	*EF1alpha*
8	*GAPDH*	*TUB*	*RAN1*	*RAN1*	*ACT*	*SAND*	*ACT*	*GAPDH*	*ACT*	*SAND*	*ACT*	*SAND*
9	*RAN1*	*CYP*	*GAPDH*	*TUB*	*GAPDH*	*GAPDH*	*GAPDH*	*SAND*	*GAPDH*	*GAPDH*	*GAPDH*	*GAPDH*

Gene expression data were analyzed using five statistical parameters in both Petunia lines. Each column refers to a gene suitability ranking computed by one statistical tool, taking into account all data of a Petunia line.

CT values were log-transformed and used as input for the NormFinder tool, which fitted this data into a mathematical model based on six independent groups corresponding to single developmental stages. Estimates for stability of gene expression are based on the comparison between inter- and intra-group variability. In the Mitchell line, the gene exhibiting the most stable level of expression was *EF1α *(stability value of 0.018) and *CYP *and *EF1α *represented the best combination (0.017). In V30, NormFinder estimated *UBQ *(0.053) as the most stably expressed gene, and *RAN1 *and *UBQ *(0.069) as the best combination of two genes.

CT values and one efficiency value for each primer pair served as input for the BestKeeper package. This program was intended to establish the best-suited standards out of the nine RG candidates, and to merge them in a normalization factor called the BestKeeper index. Because BestKeeper software is designed to determine a reliable normalization factor but not to compute the goodness of each RG independently, we took as the stability-of-expression value the coefficient of determination of each gene to the BestKeeper index. BestKeeper calculated the highest reliability for *CYP *in line Mitchell and V30 finding *GAPDH *as the least suitable gene in Mitchell and *TUB *in V30.

qBasePlus and geNorm calculate M stability values by a slightly different procedure. This parameter is defined as the average pair-wise variation in the level of transcripts from one gene with that of all other reference genes in a given group of samples; it is inversely related to expression stability. However, because the inclusion of a gene with highly variable expression can alter the estimation of the rest, geNorm (but not qBasePlus) performs a stepwise exclusion of the least stably expressed genes. Taking into account the entire dataset from Mitchell with geNorm, *RAN1 *and *SAND *were calculated to be the most stably expressed genes (M value 0.5), *GAPDH *the least (1.15). In V30, *RPS13 *and *UBQ *were calculated to be the genes of least variable expression (0.64), whereas *GAPDH *was the most variable (2.61). In terms of qBasePlus M values, *EF1α *was valued as the best gene for Mitchell (0.85) and *GAPDH *the worst (1.76); for V30, *ACT *was ranked as the most valuable gene (2.11) and *GAPDH *was the worst (3.66).

**Figure 4:**
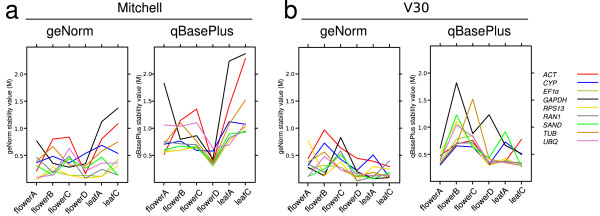
**geNorm and qBasePlus average expression stability measure values for reference genes in individual samples**. Average expression stability values (M values) are an inverse proportion measure of expression stability. GeNorm computes M values by stepwise exclusion of the least stable gene. (a) geNorm and qBasePlus output of Mitchell samples. (b) geNorm and qBasePlus output of V30 samples.

Considering each developmental stage separately, we found that M values were consistently higher in Mitchell than in V30, suggesting more variable levels of RG expression in Mitchell. Flower stage D exhibited the most stable expression pattern in both lines (Figure 4). It is noteworthy that stability of transcript levels between reproductive and vegetative modules differed in the two lines. In general, M values calculated with qBasePlus, were higher in flowers stage C and D than in leaves from Mitchell, whereas V30 showed an opposite trend. A remarkable case was *GAPDH*, with an M value four times higher in Mitchell than in V30 at leaf stage C, whereas it was three times lower in Mitchell compared to V30 at flower stage A (see Table 2).

Mean CV value, a measurement of the variation of relative quantities of RNA for a normalized reference gene, showed little difference between lines, with a value of 0.42 in Mitchell and 0.44 in V30, for data analysed as a whole.

### Determination of the number of genes for normalization

Quantification of gene expression relative to multiple reference genes implies the calculation of a normalization factor (NF) that merges data from several internal genes. Determination of the minimal number of its components is estimated by computing the pairwise variation (PV) of two sequential NFs (Vn/n+1) as the standard deviation of the logarithmically transformed NFn/NFn+1 ratios, reflecting the effect of including an additional gene [4]. If the pairwise variation value for n genes is below a cut-off of 0.15, additional genes are considered not to improve normalization. The number of genes required for normalization was determined to be two for both Mitchell and V30, except when either different floral developmental stages or vegetative and reproductive stages were mixed (see Table 2).

**Figure 5:**
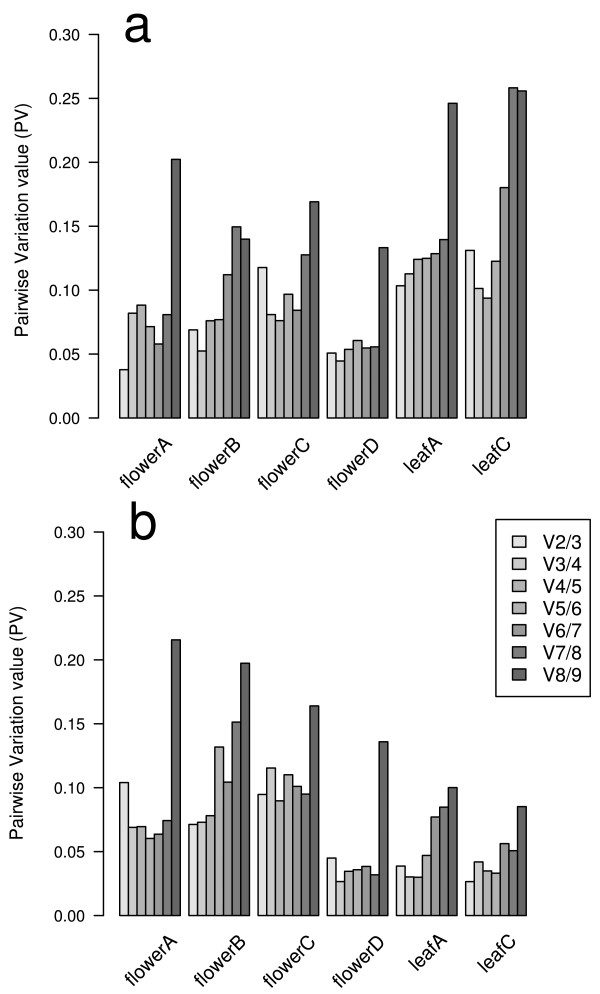
**Minimum number of genes necessary for reliable and accurate normalization**. GeNorm pairwise variation values (PV values) are computed by an algorithm which measures pairwise variation (Vn/n + 1) between two sequential normalization factors NFn and NFn + 1, where n is the number of genes involved in the normalization factor. (a) refers to Mitchell line and (b) to V30.

The PV values showed the same trend as that seen for stability measurements, i.e., the developmental stage with the lowest average PV was flower stage D, both in Mitchell and V30. In contrast, gene expression in leaves of Mitchell showed more variability, with higher PV values, than those of V30 (Figure 5).

### Consensus list of similarities between lines

**Figure 6:**
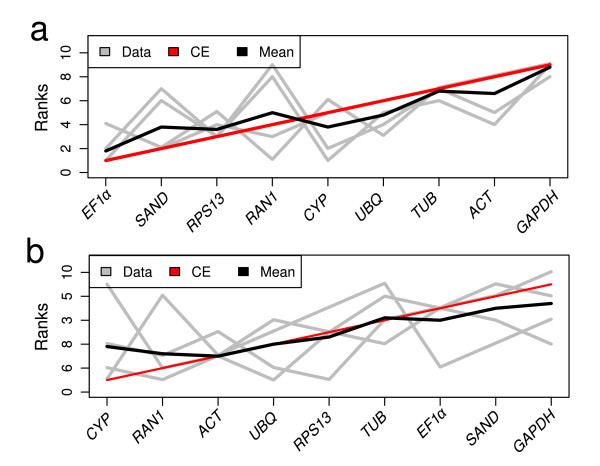
**Rank aggregation of gene lists using the Monte Carlo algorithm**. Visual representation of rank aggregation using Monte Carlo algorithm with the Spearman footrule distances. (a) refers to Mitchell line and (b) to V30. The solution of the rank aggregation is shown in a plot in which genes are ordered based on their rank position according to each stability measurement (grey lines). Mean rank position of each gene is shown in black, as well the model computed by the Monte Carlo algorithm (red line).

The different software programs used to determine gene suitability for normalization of gene expression give slightly different results and statistical stability values for each gene. We arranged the internal genes in five lists according to the rank positions generated by each of the five statistical approaches, M values by geNorm and qBasePlus, NormFinder stability value, coefficient of determination to BestKeeper and CV of qBasePlus. These lists were used to create an aggregate order, with the aim of obtaining an optimal list of genes for each Petunia line. The results of the merged data revealed that the most adequate of the genes tested for normalization in Mitchell are *EF1α*, *SAND *and *RPS13*; the three showing the lowest reliability are *TUB*, *ACT *and *GAPDH *(Figure 6A and 6B). For V30, the best candidate genes are *CYP*, *RAN1 *and *ACT*, while the three lowest ranking are *EF1α*, *SAND *and *GAPDH*. Thus none of the genes found as highly reliable coincide between the lines. Despite of that, *GAPDH *was highly unstable in both lines.

## Discussion

### Identification of robust normalization genes for Petunia

We have attempted to identify a set of genes suitable for normalization of transcript levels in *P. hybrida*. Since several Petunia lines are used for research, we based this work on two that are extensively used for different purposes. In an effort to reflect different growth environments typical of distinct lab setups, plants of each line were grown in a set of conditions, differing in photoperiod, thermoperiod and growth substrate between lines (see methods). RNA was isolated using different RNA extraction kits, and amplifications were carried out using different reagents and PCR machines. The experimental design aimed to maximize potential variability in transcript abundance for the putative RG under study. Highly contrasting results would suggest that every laboratory do a pilot experiment to identify genes suitable for use in normalization; similar results between the two systems would point to a set of genes reliable for broad application, minimally for the lines and developmental stages described.

Our findings in terms of line-associated variability were not in accordance with the results from a soybean study comparing different cultivars. Results of that study suggested no highly relevant cultivar influence on RG suitability [16]. A similar study has been reported in coffee, for which average M stability values for leaves from different cultivars were lower than that for different organs of a single cultivar. Our result suggests that there are differences in gene expression between same tissues from different lines as well as different tissues from the same line.

### Noise in gene expression patterns

Development of petals, like that of many tissues and organs in Petunia, is characterized by a spatial and temporal gradient of cell division that is eventually replaced by cell expansion [28]. However the experiments described here used whole flower tissues including full petals along with sepals, stamens and carpels. This imposes a general requirement that any gene emerging as robust be differentially regulated to a huge extent neither in the various tissues analyzed together nor in these tissues at different stages of maturation. One interesting aspect of our findings was the identification of flower stage C as a particularly noisy developmental stage compared to early or fully developed flowers. The transition between cell division and expansion in petals, or other flower tissues during this developmental stage, might explain the increased noise. An alternative non-exclusive explanation is that the intermediate stages of flower development are generally less tightly defined than the open flower stage.

Leaf development similarly consists of cell growth followed with cell expansion [29]. However, an important difference between floral and leaf development is that leaves perform their essential function, e.g., photosynthesis, from a very early stage such that developing leaf tissue is always a mixture of at least three processes: growth, cell morphogenesis and differentiated cell function. This combination of processes might account for the increased gene expression noise observed.

### Number of genes required for normalization of gene expression in Petunia

Gathering data from several RG into a normalization factor is currently an accepted method of accurate relative quantification of gene expression [30]. Moreover, this method has been statistically and empirically validated [13,31]. Ideally the number of genes required should be low enough to make experimental procedures affordable, and high enough to merit confidence in the conclusions. The PV value obtained for both Mitchell and V30 was very low. Although the value tended to be higher in Mitchell, the number of genes deemed necessary for normalization was the same for both lines: using the proposed cut-off of 0.15 and comparing single developmental stages, the required number was two for Mitchell and V30. The requirement for only two genes is low compared to the results reported for other phylogenetically related species [10,15,32] and will require significantly less work than the previously suggested minimum of three genes [4].

### Data mining strategies and consensus list of genes for normalization

The present research aims to identify the control genes best suited for use in gene expression studies in several organs of two Petunia lines. The candidate RG combined classical and recently identified genes. Since each software package can introduce bias, we employed several tools in our analysis. As discussed by other authors, geNorm bases its stability measurement on pairwise comparisons of relative expression quantities of all the panel of genes in the material of interest requiring a suite of non-coregulated RG [6]. BestKeeper and NormFinder examine primarily CT values, whereas qBasePlus and geNorm evaluate RQ, a consequence of which is that PCR efficiency dissimilarities can affect stability measurements [16]. Nevertheless, some of these algorithms are intrinsically biased because they assume that data are normally distributed. For instance BestKeeper is based on Pearson correlation analysis, which requires normally distributed and variance homogeneous data. The author described this problem and suggested further versions of the software in which Spearman and Kendall Tau correlation should be used [5]. However, those versions are currently not available.

Our plant material diverged in the variability of statistical outputs amongst lines. V30 showed a high variability in terms of raw expression data (CT values) and low in terms of expression stability measurements, whereas Mitchell showed the opposite responses. Our global analysis merged different statistics, some of which are CT-based and others RQ-based, with the aim of counteracting this biasing influence.

Summarizing the results of our entire dataset analysis, geNorm recommended use of *RAN1 *and *SAND *genes for Mitchell and *RPS13 *and *UBQ *for V30 and discouraged use of *GAPDH *for both lines. Non-suitability of *GAPDH *has been described by several authors [33,34]. Regarding to Solanaceae, its unsuitability has been confirmed in tomato [15] but it was selected as a stable RG in coffee [35]. Due to its sequential exclusion of the least stable gene in the M value calculus algorithm, geNorm M values can differ from those of qBasePlus. qBasePlus corresponded with geNorm, evaluating *EF1α *as the most reliable gene in line Mitchell but differed in line V30, recommending *ACT *as the best candidate. *EF1α *suitability has been confirmed in potato during biotic and abiotic stress [10], atlantic salmon [36] and several developmental stages of *Xenopus laevis *[37]. Expression of *ACT *genes differs depending on the family member. *ACT2*/7 has been reported as a stably expressed gene whereas *ACT11 *was reported as unstable [38,39]. It is worth noting that the *ACT *gene used in this study corresponds to an *ACT11*.

## Conclusions

Altogether, there were strong similarities between the different programs but the coincidence in assigning best and worst genes was not absolute. The fact that each program identified slightly different genes as best suited for normalization prompted us to merge the data in an unsupervised way and giving identical weight to the output of the different programs. We used the RankAggreg program for this purpose. Our results show that *GAPDH *was the worst gene to use in normalization in both lines. In contrast, the suggested genes did not coincide and were *EF1α *and *SAND *in Mitchell, whilst *CYP *and *RAN1 *were the genes of choice in V30. In conclusion, we provide a list of genes in discrete developmental stages that show M values below 0.5 (Table 2) [4]. A normalization factor including two genes should be enough for reliable quantification. Nevertheless we propose a reference gene stability test when performing gene expression studies in Petunia.

## Methods

### Plant material

*Petunia hybrida *lines Mitchell and V30 were grown in growth chambers. Mitchell plants were grown on ED73 + Optifer (Patzer) under a 10 h light/14 h dark cycle, with a constant temperature of 22°C (60% humidity).

V30 plants were germinated in vermiculite and grown in a vermiculite-perlite-turf-coconut fiber mixture (2:1:2:2). Plants were kept under a long day photoperiod (16L: 8 D) with 25°C in L and 18°C in D.

Flowers were classified into four developmental stages: flower buds (stage A, 1-1.5 cm), elongated buds (stage B, 2,5-3 cm), pre-anthesis (stage C, 3.5 -4.5 cm) and fully opened flowers shortly before anthesis (stage D) according to Cnudde et al. [40]. Leaves were harvested at two different stages, stage A corresponded to young, small leaves and stage C to fully expanded ones. Three independent samples of each of the developmental stages of flowers and leaves were taken.

### RNA isolation and cDNA synthesis

#### Mitchell material

Total RNA was isolated from 100 mg homogenized plant material using an RNeasy Mini Kit (Qiagen, Hilden, Germany). Putative genomic DNA contamination was eliminated by treatment with recombinant DNase I (Qiagen) as recommended by the vendor. RNA concentration and purity was estimated from the ratio of absorbance readings at 260 and 280 nm and the RNA integrity was tested by gel electrophoresis. cDNA synthesis was performed using M-MLV reverse transcriptase (Promega, Mannheim, Germany) starting with 1 μg of total RNA in a volume of 20 μL with oligo(dT)19 primer at 42°C for 50 min.

#### V30 material

Samples were homogenized in liquid nitrogen with a mortar and pestle. Total RNA was isolated using the NucleoSpin^® ^RNA Plant (Macherey-Nagel, Düren, Germany) according to the manufacturer's protocol. This RNA isolation kit contains DNaseI in the extraction buffer, added to the column once RNA is bound to the spin column. RNA was measured by photometry at 260 nm and quality-controlled on denaturing agarose gels. Total RNA (0.8 μg) was transcribed using the SuperScript^® ^III (Invitrogen Corp., Carlsbad, CA) and oligodT20 employing 10 μL 2× RT reaction mix, 2 μL RT enzyme mix and 8 μL RNA. Reverse transcription was performed on a GeneAmp Perkin-Elmer 9700 thermocycler (Perkin Elmer, Norwalk, CT, USA) by using the following programme: 10 min at 25°C, 30 min at 50°C and 5 min at 85°C; addition of 1 u of Escherichia coli RNAse H, and incubation for 2 h at 15°C.

### PCR optimisation

We selected nine genes to be tested as reference transcripts (*ACT*, *CYP*, *EF1α*, *GAPDH*, *RAN2*, *RPS13*, *SAND *and *UBQ*) based on previous descriptions (see below) (Table 1). PCR conditions were optimised using cDNA from leaves (stage A) in a Robocycler gradient 96 (Stratagene, La Jolla, CA) and GoTaq^® ^Flexi DNA polymerase (Promega) in a 25 μL reaction containing: 2 μL of cDNA, 2 mM MgCl2, 0.2 mM each dNTP, 0.4 μL of each primer and 1.25 U enzyme.

### Real-time PCR

#### Mitchell

Real-time PCR was performed in an Mx 3005P QPCR system (Stratagene, La Jolla, CA) using a SYBR Green based PCR assay (with ROX as the optional reference dye; Power SYBR Green PCR Mastermix, Applied Biosystems, Foster City, CA). A master mix containing enzymes and primers was added individually per well. Each reaction mix containing a 15 ng RNA equivalent of cDNA and 1 pM gene-specific primers (Tab. 3) was subjected to the following protocol: 95°C for 10 min followed by 50 cycles of 95°C for 30 sec, 60°C for 1 min and 72°C for 30 sec, and a subsequent standard dissociation protocol. As a control for genomic DNA contamination, 15 ng of total non-transcribed RNA was used under the same conditions as described above. All assays were performed in three technical replicates, as well three biological replicates.

#### V30

Reactions were carried out with the SYBR Premix Ex Taq^® ^(TaKaRa Biotechnology, Dalian, Jiangsu, China) in a Rotor-Gene 2000 thermocycler (Corbett Research, Sydney, Australia) and analysed with Rotor-Gene analysis software v. 6.0 as described before [41] with the following modifications: Reaction profiles used were 40 cycles of 95°C for 30 s, 55°C or 60°C for 20 s, 72°C for 15 s, and 80°C for 15 s, followed by melting at 50-95°C employing the following protocol: 2 μL RNA equivalent of cDNA, 7.5 μL SYBR Premix Ex Taq 2×, 0.36 μL of each primer at 10 μM and 4.78 μL distilled water. Annealing temperature was 55°C (*TUB*, *CYP*, *ACT*, *EF1α*, *GAPDH*, *and SAND*) or 60°C (*RPS13*, *UBQ*, *RAN1*) according to the previous optimisation. In order to reduce pipetting variability, we performed reaction batches containing primer pairs, and templates were added in the end. We performed three technical replicates for each reaction and non-template controls, as well three biological replicates.

### Bioinformatics and statistical analysis

Data analysis strategy is described in detail in results. Reaction efficiency calculus was done using the amplification curve fluorescence, analyzing each tube separately as described by Liu and Saint (2002) [42]. It was calculated as follows: Efficiency = F(n)/F(n-1), in which n is defined as the 20% value of the fluorescence at the maximum of the second derivative curve. Curve was defined by one measure in each amplification cycle. We used only the exponential phase of the amplification reaction. Software packages included geNorm v3.4, the excel add-in of NormFinder v0.953, BestKeeper v1 and qBasePlus v1.2. Other statistical procedures were performed with the R program http://www.R-project.org, v2.7.1 with the packages stats v2.7.1, multcompView v0.1-0 and RankAggreg v0.3-1[27].

## Abbreviations

*ACT*: *Actin 11*; CT: cycle threshold; CV: coefficient of variation; *CYP: Cyclophilin*; *EF1α: Elongation factor 1-alpha; GAPDH: Glyceraldehyde-3-phosphate dehydrogenase*; qPCR: quantitative PCR; *RAN1: GTP-binding nuclear protein; *RG: reference genes; *RPS13: Ribosomal protein S13*; RQ: Relative quantity; *SAND: SAND family protein; TUB: b-Tubulin 6 chain; UBQ: Polyubiquitin*.

## Authors' contributions

IM, BH, JW and MEC designed the experiments. IM and SL performed the experiments. IM performed data analysis and table and figure drawing. MEC wrote the first draft, and IM, BH, SL, JW and MEC corrected and approved the manuscript. JW, BH and MEC wrote grant applications.

## Supplementary Material

Additional File 1 Mitchell line. Click here for file

Additional File 2 Supplemental tables. Click here for file

Additional File 3 Supplemental tables. Click here for file

Additional File 4 Supplemental tables. Click here for file
