# Supplementary material for: Validation of reference genes for quantitative real-time PCR during leaf and flower development in Petunia hybrida
Source: BMC Plant Biol. 2010 Jan 7;10:4. doi: 10.1186/1471-2229-10-4 (PMC2827423; doi:10.1186/1471-2229-10-4)
Supplement: Additional File 1 — Mitchell line. [file 1471-2229-10-4-S1.RTF]

Mitchell line

Table: Shapiro-Wilks normality test of CT values. Significance cut-off: p<0.05
Gene	ACT	CYP	EF1alpha	GAPDH	RAN3	RPS13	SAND	TUB	UBQ	
p-value	0.6016
	0.002345
	0.00435
	5.425e-05
	5.465e-08	0.2225
	7.5e-05
	 0.003274
	0.2307
	
W	0.982	0.9239	0.9308	0.8761	0.7571	0.971	0.8806	0.9276	0.9709	

Table: Kruskal-Wallis rank sum test of CT values. Significance cut-off: p<0.05.

Gene	ACT	CYP	EF1alpha	GAPDH	RAN3	RPS13	SAND	TUB	UBQ	
p-value	0.1292
	0.04285	0.01742	0.0184	0.0007533	0.001030	0.04381	0.04081	0.005163	
Kruskal-Wallis chi squared	8.5325	11.4682	13.7305	13.5949	21.1669	20.4474	11.4109	11.5933	17.1844	
df	5	5	5	5	5	5	5	5	5	

Table: Pairwise comparisons using Wilcoxon rank sum test of significantly different CT values. Significance cut-off: p<0.05 with Bonferroni's correction. For each gene, same letters represent samples which are not significantly different.
organ	ACT	CYP	EF1alpha	GAPDH	RAN3	RPS13	SAND	TUB	UBQ	
FlowerA	a	a	a	a	ab	a	a	a	ab	
FlowerB	a	a	a	a	ab	a	a	a	ab	
FlowerC	a	a	a	a	a	a	a	a	ab	
FlowerD	a	a	a	a	a	b	a	a	a	
LeafA	a	a	a	a	b	ab	a	a	b	
LeafC	a	a	a	a	ab	ab	a	a	ab	

Pairwise Wilcoxon tests results (computed with Bonferroni's correction):

Data:  ACT and organ 

	FlowerA	FlowerB	FlowerC	FlowerD	LeafA
	
FlowerB	1.00	- 	-	-	-    
	
FlowerC	0.12	1.00	-	-	-    
	
FlowerD	0.28	1.00	1.00	-	-    
	
LeafA	1.00	1.00	1.00	1.00	-    
	
LeafC	1.00	1.00	0.75	1.00	1.00 
	


Data: CYP and organ	

	FlowerA	FlowerB	FlowerC	FlowerD	LeafA	
FlowerB	1.00	-	-	-	-	
FlowerC	0.51	1.00	-	-	-	
FlowerD	1.00	0.75	0.12	-	-	
LeafA	1.00	0.70	1.00	1.00	-	
LeafC	1.00	1.00	0.23	1.00	1.00	

	

	

Data: EF1alpha and organ	

	FlowerA	FlowerB	FlowerC	FlowerD	LeafA
	
FlowerB	1.00	-	-	-	-	
FlowerC	1.00	1.00	-	-	-	
FlowerD	0.60	0.37	0.07	-	-	
LeafA	0.70	1.00	1.00	1.00	-	
LeafC	1.00	0.57	0.63	1.00	1.00	
	


Data: GAPDH and organ	

	FlowerA	FlowerB	FlowerC	FlowerD	LeafA	
FlowerB	1.0000	-	-	-	-	
FlowerC	0.4079	1.0000	-	-	-	
FlowerD	0.0074	0.7546	0.9471	-	-	
LeafA	1.0000	1.0000	1.0000	0.3097	-	
LeafC	1.0000	1.0000	1.0000	0.3665	1.0000
	


Data: RAN3 and organ	

	FlowerA	FlowerB	FlowerC	FlowerD	LeafA
	
FlowerB	1.000	-	-	-	-	
FlowerC	0.568	0.061	-	-	-	
FlowerD	1.000	0.159	1.000	-	-	
LeafA	0.274	1.000	0.015	0.037	-	
LeafC	1.000	1.000	0.156	0.510	1.000
	

Data: RPS13 and organ	

	FlowerA	FlowerB	FlowerC	FlowerD	LeafA	
FlowerB	1.00000	-	-	-	-	
FlowerC	1.00000	1.00000	-	-	-	
FlowerD	0.01851	0.03456	0.00062	-	-	
LeafA	1.00000	1.00000	1.00000	1.00000	-	
LeafC	1.00000	1.00000	0.05985	0.05985	1.00000
	


Data: SAND and organ	

	FlowerA	FlowerB	FlowerC	FlowerD	LeafA
	
FlowerB	0.159	-	-	-	-	
FlowerC	0.213	1.000	-	-	-	
FlowerD	1.000	1.000	1.000	-	-	
LeafA	1.000	1.000	1.000	1.000	-	
LeafC	0.093	1.000	1.000	0.510	1.000
	


Data: TUB and organ	

	FlowerA	FlowerB	FlowerC	FlowerD	LeafA	
FlowerB	1.0000	-	-	-	-	
FlowerC	1.0000	1.0000	-	-	-	
FlowerD	0.2559	1.0000	0.0061	-	-	
LeafA	0.8885	1.0000	1.0000	1.0000	-	
LeafC	1.0000	1.0000	1.0000	1.0000	1.0000
	


Data: UBQ and organ	

	FlowerA	FlowerB	FlowerC	FlowerD	LeafA	
FlowerB	1.0000	-	-	-	-	
FlowerC	1.0000	1.0000	-	-	-	
FlowerD	0.0599	1.0000	1.0000	-	-	
LeafA	1.0000	0.5688	0.1666	0.0086	-	
LeafC	1.0000	1.0000	1.0000	0.5998	0.2412
	

V30 line
Table: Shapiro-Wilks normality test of CT values. Significance cut-off: p<0.05.
Gene	ACT	CYP	EF1alpha	GAPDH	RAN3	RPS13	SAND	TUB	UBQ	
p-value	 0.001487	 1.089e-09	 0.0009683	 0.001065	 0.003819	 1.109e-05	 0.000359	 1.114e-05	 6.962e-05	
W	0.9199	0.7736	0.9136	0.9161	0.9305	0.85	0.903	0.8474	0.8816	

Table: Kruskal-Wallis rank sum test of CT values. Significance cut-off: p<0.05.
Gene	ACT	CYP	EF1alpha	GAPDH	RAN3	RPS13	SAND	TUB	UBQ	
p-value	3.213e-07	2.633e-08	1.439e-08	3.047e-09	6.122e-08	3.812e-08	1.097e-09	1.945e-07	1.716e-08	
Kruskal-Wallis chi-squared	38.3476
	43.7254	45.0175	48.326	41.9173	42.9332	 50.4951	39.4308	44.642	
df	5	5	5	5	5	5	5	5	5	

Table: Pairwise comparisons using Wilcoxon rank sum test of significantly different CT values. Significance cut-off: p<0.05 with Bonferroni's correction. For each gene, same letters represent samples which are not significantly different.

organ	ACT	CYP	EF1alpha	GAPDH	RAN3	RPS13	SAND	TUB	UBQ	
FlowerA	a	a	a	a	a	a	a	a	a	
FlowerB	a	a	b	b	b	a	b	ab	b	
FlowerC	b	b	b	b	c	b	c	ac	c	
FlowerD	c	c	c	a	d	c	d	c	d	
LeafA	ab	bc	cd	c	acd	bc	e	c	cd	
LeafC	a	bc	ad	d	c	c	e	b	c	


Pairwise Wilcoxon tests (computed with Bonferroni's correction) 

Data: ACT and organ	

	FlowerA	FlowerB	FlowerC	FlowerD	LeafA	
FlowerB	1.00000	-	-	-	-	
FlowerC	0.00614	0.00062	-	-	-	
FlowerD	0.00614	0.00062	0.00062	-	-	
LeafA	0.25592	0.21288	1.00000	0.00740	-	
LeafC	1.00000	1.00000	0.00062	0.00062	1.00000
	


Data: CYP and organ	

	FlowerA	FlowerB	FlowerC	FlowerD	LeafA	
FlowerB	1.0000	-	-	-	-	
FlowerC	0.0011	0.0018	-	-	-	
FlowerD	0.0366	0.0307	0.0368	-	-	
LeafA	0.0011	0.0011	0.6377	1.0000	-	
LeafC	0.0162	0.0196	0.1888	0.5393	1.0000
	

	

Data: EF1alpha and organ	

	FlowerA	FlowerB	FlowerC	FlowerD	LeafA	
FlowerB	0.00062	-	-	-	-	
FlowerC	0.00123	1.00000	-	-	-	
FlowerD	0.00062	0.00062	0.00123	-	-	
LeafA	0.00740	0.00062	0.00123	1.00000	-	
LeafC	0.05985	0.00062	0.00123	0.00062	1.00000
	


Data: GAPDH and organ	

	FlowerA	FlowerB	FlowerC	FlowerD	LeafA	
FlowerB	0.00062	-	-	-	-	
FlowerC	0.00614	1.00000	-	-	-	
FlowerD	1.00000	0.00062	0.00614	-	-	
LeafA	0.00062	0.00432	0.00614	0.00062	-	
LeafC	0.00062	0.00062	0.00614	0.00432	0.00062	


Data: RAN1 and organ	

	FlowerA	FlowerB	FlowerC	FlowerD	LeafA	
FlowerB	0.01581	-	-	-	-	
FlowerC	0.00602	0.00614	-	-	-	
FlowerD	0.00602	0.00614	0.00062	-	-	
LeafA	0.09092	0.02193	1.00000	1.00000	-	
LeafC	0.00602	0.00614	0.05985	0.00062	1.00000
	


Data: RPS13 and organ	

	FlowerA	FlowerB	FlowerC	FlowerD	LeafA	
FlowerB	1.00000	-	-	-	-	
FlowerC	0.01536	0.00062	-	-	-	
FlowerD	0.01536	0.00062	0.00062	-	-	
LeafA	0.01536	0.00062	0.36652	1.00000	-	
LeafC	0.01536	0.00062	0.00062	0.08080	0.28137
	


Data: SAND and organ	

	FlowerA	FlowerB	FlowerC	FlowerD	LeafA	
FlowerB	0.00062	-	-	-	-	
FlowerC	0.00062	0.00432	-	-	-	
FlowerD	0.00062	0.00062	0.00062	-	-	
LeafA	0.00614	0.00614	0.00614	0.00614	-	
LeafC	0.00062	0.00062	0.00062	0.00062	0.32425
	


Data: TUB and organ	

	FlowerA	FlowerB	FlowerC	FlowerD	LeafA	
FlowerB	1.00000	-	-	-	-	
FlowerC	0.22830	0.28137	-	-	-	
FlowerD	0.02169	0.01536	1.00000	-	-	
LeafA	0.00123	0.00062	0.13731	1.00000	-	
LeafC	0.00123	0.05985	0.00062	0.01536	0.00062
	


Data: UBQ and organ	

	FlowerA	FlowerB	FlowerC	FlowerD	LeafA	
FlowerB	0.01384	-	-	-	-	
FlowerC	0.00606	0.00606	-	-	-	
FlowerD	0.00614	0.00614	0.00602	-	-	
LeafA	0.00062	0.00062	0.69736	1.00000	-	
LeafC	0.00614	0.00614	1.00000	0.00610	1.00000

	
